# Antibiofilm sustainable strategies: pomegranate extract from agri-food waste as a natural antifungal against emerging *Candida* pathogens

**DOI:** 10.3389/fmicb.2025.1724685

**Published:** 2025-11-19

**Authors:** Daniela Sateriale, Giuseppina Forgione, Sharon Raucci, Abdul Jabbar, Roberta Imperatore, Chiara Germinario, Chiara Pagliuca, Roberta Colicchio, Mariateresa Vitiello, Mariano Mercurio, Marina Paolucci, Paola Salvatore, Caterina Pagliarulo

**Affiliations:** 1 Department of Science and Technology, University of Sannio, Benevento, Italy; 2 Department of Chemical Engineering, Materials and Industrial Production, University of Naples Federico II, Naples, Italy; 3 Department of Molecular Medicine and Medical Biotechnologies, University of Naples Federico II, Naples, Italy; 4 CEINGE-Biotecnologie Avanzate Franco Salvatore s.c.ar.l., Naples, Italy

**Keywords:** pomegranate peel extract, *Candida* spp., antimycotic activity, antigerminative properties, biofilm inhibition, biofilm eradication

## Abstract

**Introduction:**

*Candida* spp. infections are a leading cause of morbidity, particularly among immunocompromised and hospitalized patients. Their pathogenicity is driven by multiple virulence factors, including dimorphic transitions, germ tube formation, and biofilm development. Biofilms enhance resistance to antifungal agents and immune defenses, making biofilm-associated *Candida* infections a significant clinical challenge. There is an urgent need for effective and sustainable therapies, with agri-food waste emerging as a promising source of bioactive compounds.

**Methods:**

This in vitro study evaluated the antifungal activity of a polyphenol-rich hydroethanolic pomegranate (*Punica granatum* L.) peel extract against clinical isolates of *Candida albicans, C. glabrata*, and *C. parapsilosis*. The extract was tested for its antimycotic effects using qualitative and quantitative assays, as well as its ability to inhibit germ tube formation and biofilm development, including adhesion and disruption of pre-formed biofilms on plastic surfaces commonly used in medical devices.

**Results:**

The pomegranate peel extract exhibited significant antifungal activity, demonstrating both fungistatic and fungicidal effects across all tested strains. The extract interfered with fungal membrane permeability and inhibited key virulence mechanisms, including germ tube formation and biofilm development. Notably, it reduced adhesion and disrupted established biofilms.

**Discussion:**

These findings highlight the anti-*Candida* potential of pomegranate peel extract, targeting multiple virulence determinants. The results support the development of alternative therapeutic strategies against emerging biofilm-related fungal infections, leveraging sustainable approaches based on local agri-food byproducts.

## Graphical Abstract

**Figure:**
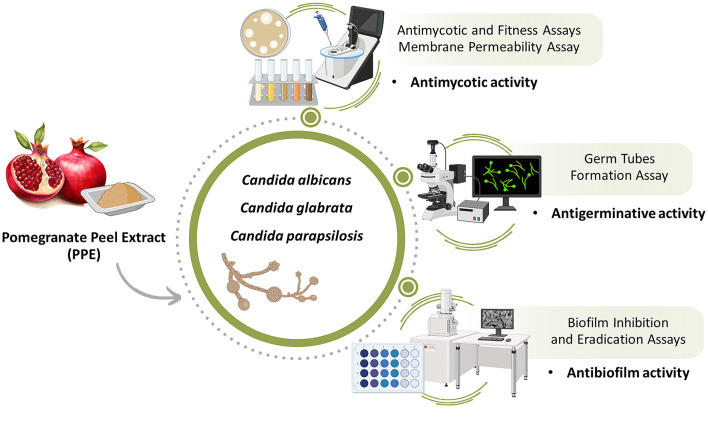

## 1 Introduction

Historically, fungal infections have been regarded as a relatively uncommon cause of clinically significant disease when compared to bacterial and viral infections (Richardson, 2005). Throughout the latter half of the 20th century, there has been a notable shift in trends, marked by a significant rise in the incidence of fungal infections among humans (Seagle et al., 2021). Fungi represent a diverse group of microorganisms that occupy a variety of ecological niches. They are commonly recognized as saprophytic commensals inhabiting the skin and mucous membranes of mammals, including humans. Under typical circumstances, the host immune system effectively regulates the proliferation of these microorganisms. However, the increasing prevalence of opportunistic fungal infections has emerged as a significant concern. The escalation in fungal infections is largely attributed to the increased number of immunocompromised patients and the widespread use of medical practices such as invasive surgery, immunosuppressive therapies, and broad-spectrum antimicrobials (Kojic and Darouiche, 2004; Casadevall, 2018). Among the pathogenic fungi, members of the *Candida* genus are the most frequent cause of invasive human fungal diseases (Macias-Paz et al., 2023). The *Candida* genus includes over 150 species, although only a few are implicated in human candidiasis. *Candida albicans* is the most commonly encountered species in both healthy and diseased individuals (Calderone and Fonzi, 2001). *C. albicans* typically colonizes the oral cavity, skin, gastrointestinal tract and vagina. In individuals with a healthy immune system, *C. albicans* is typically benign, maintaining a balanced relationship with other microorganisms in the local microbiota. However, disruptions in the host microbiota, changes in the immune response, or alterations in the local environment can promote the overgrowth of *C. albicans*, leading to infections. This shift from mutualism to pathogenicity may result in infections ranging from superficial mucosal and skin conditions, such as thrush, vaginal yeast candidiasis, and diaper rashes, to more severe disseminated infections that spread through the bloodstream, with high mortality rates, sometimes approaching 40%, particularly in vulnerable patient populations (Nobile and Johnson, 2015). However, over the past decades, with the increasing use of antimycotic agents and advanced diagnostic techniques, a significant rise in infections caused by non-*C. albicans* species (NCAC) has been observed (Kauffman et al., 2000). These species, such as *C. glabrata, C. parapsilosis*, and *C. auris* are emerging as important opportunistic agents, further complicating the management of multidrug-resistant fungal pathogens (Caceres et al., 2019; Kim et al., 2024). NCAC infections are especially serious in immunocompromised individuals and healthy people with implanted medical devices (Calderone, 2002; Nobile and Johnson, 2015). Notably, many of these NCAC species exhibit higher intrinsic resistance to antimycotic drugs, which may promote their persistence in mixed infections treated with conventional antimycotic agents (González et al., 2008).

The pathogenicity of *Candida* species is multifactorial, involving host and microorganism-specific factors, such as phenotypic switching, metabolic plasticity, extracellular hydrolytic enzymes, and biofilm formation (érez and Johnson, 2013). Biofilms, in particular, contribute significantly to the persistence of infections, as they create a physical barrier that limits the penetration of antimycotic drugs, thereby protecting embedded microbial cells from both therapeutic treatments and the host's immune system (Chellat et al., 2016). In the case of *Candida* spp., there is a strong correlation between biofilm formation and the germination process. Germination is crucial in fungal development, as stress-resistant spores transition from a dormant state to an active, vegetative form. This transition is essential for *Candida* to colonize new environments and is directly linked to its ability to form biofilms, which further promote its survival and virulence (Wyatt et al., 2013). Furthermore, germination induces a crucial morphological change that significantly enhances the yeast's ability to firmly adhere to various human mucosal surfaces. This contributes to increase pathogenicity and the ability of *Candida* to cause resistant infections (Matsumoto et al., 2013).

Antimycotic resistance represents a significant clinical challenge in the treatment of invasive fungal infections, primarily due to the limited arsenal of systemically available antimycotic agents. Additionally, current antimycotic drugs may be restricted by serious adverse effects and toxicities, which prevent their prolonged use or require dosage adjustments (Wiederhold, 2017). Azole resistance is of particular concern, especially for NCAC, due to the increased incidence of infections caused by these species and the high prevalence of resistance to these commonly used antimycotic, often necessitating higher doses (Yapar, 2014). However, at elevated concentrations, azoles can be toxic, leading to side effects such as liver damage, endocrine disruptions, and gastrointestinal issues. The growing issue of resistance and the toxicity of existing antimycotic drugs underscore the need for new, selective, and less toxic alternatives.

In recent years, plant-derived extracts have gained attention as potential antimycotic agents (Vidyasagar, 2016; Pagliarulo et al., 2018; Sateriale et al., 2019; Esmaeili et al., 2025). A key benefit of exploring plant-based antimicrobials is the potential to extract bioactive molecules from agri-food waste. Utilizing these by-products not only offers a sustainable approach against fungal infections but also adds value to otherwise underutilized materials. Pomegranate peel (*Punica granatum* L.), specifically, has been identified as a rich source of bioactive phytochemicals, including polyphenols, flavonoids, tannins, and alkaloids. Among these, punicalagins, ellagic acid, and anthocyanins are the most extensively studied for their antioxidant, anticancer, antibacterial, and antimycotic properties (Bassiri-Jahromi et al., 2015; Singh et al., 2018; Forgione et al., 2024). Punicalagins, in particular, have demonstrated notable antimycotic activity, effectively inhibiting the growth of various pathogenic fungi, including *Candida* species (Abd-Elmonsef, 2021; Celiksoy et al., 2022). Recent studies have also highlighted the ability of natural extracts to inhibit biofilm formation, a critical factor in the persistence of fungal infections, offering new avenues for fighting biofilm-associated infections (Asma et al., 2022; Shamim et al., 2023). Furthermore, recent studies have shown that hydroethanolic extracts of pomegranate peel exhibit no cytotoxic effects on several human cell lines, underscoring their potential as a safer alternative for therapeutic applications (Scaglione et al., 2024). This non-cytotoxicity is crucial for ensuring the safety of long-term use in clinical settings, offering a promising option for patients, especially those with compromised immune systems, who are at greater risk from traditional antimycotic treatments. The ability to combine efficacy against infections with a favorable safety profile positions pomegranate peel extracts as a valuable candidate for the development of new, more sustainable therapeutic strategies, especially against drug-resistant fungal infections.

Given the increasing prevalence of *Candida* infections, particularly those associated with biofilm formation and the growing threat of antimicrobial resistance, there is an urgent need for innovative therapeutic solutions. This study addresses this need by exploring the *in vitro* antimycotic properties of polyphenolic extracts from pomegranate peel (*Punica granatum* L.), focusing on their potential to fight resistant fungal infections. This research adopts a multifaceted approach, evaluating antimycotic, antibiofilm, and antigerminative properties of pomegranate peel extracts against a range of *Candida* species, including *C. albicans*, as well as emerging species such as *C. glabrata* and *C. parapsilosis*. This study presents new perspectives on the use of plant waste, in particular pomegranate peel, as a sustainable and valuable resource for developing alternative antifungal treatments. By demonstrating its ability to combat biofilm-associated resistant fungal infections, the findings highlight the potential of pomegranate peel extract for future application in non-toxic, plant-based antifungal therapies.

## 2 Materials and methods

### 2.1 Pomegranate peel extract (PPE)

The hydroethanolic polyphenolic extract used in this study was obtained from samples of dried peel of pomegranate fruits, harvested from plants growing in the rural areas of the Campania region in Italy, specifically in the Avellino area (40.9N, 14.7E, data available on the Global Biodiversity Information Facility platform, GBIF, https://www.gbif.org/occurrence/3949347683), using a solid-liquid solvent extraction method, according to the protocols described by Pagliarulo et al. (2016) and Sateriale et al. (2020a). Briefly, pomegranate peel powder was homogenized in an extraction solution composed of ethanol (≥99.8%, CAS 64-17-5, Sigma-Aldrich, Merck, Darmstadt, Germany) and distilled water, for 30 min at 25 ± 2 °C in the dark; dilution ratio of 1:10 (w/v). After centrifugation at 10,000 rpm for 15 min (Centrifuge 5,804 R, Eppendorf, Milan, Italy), the supernatant was filtered using a vacuum filtration unit with a 0.45 μm porosity membrane (Sterilcup^®^ S2HVU02RE Filtration System, Merck-Millipore, Darmstadt, Germany). The extract will be mentioned in the manuscript as PPE (Pomegranate Peel Extract).

The filtered extract was analyzed by Folin–Ciocalteu colorimetric assay (Singleton and Rossi, 1965) for total polyphenol content (TPC), espressed in gallic acid equivalents per sample (mg GAE g^−1^), based on the calibration curve (0–200 mg L^−1^) of gallic acid (Sigma-Aldrich Chemie, Steinheim, Germany). The detailed characterization of the phenolic compounds was performed using high-performance liquid chromatography (HPLC) (HP 1100, Agilent Technologies, Palo Alto, California, USA) and matrix-assisted laser desorption/ionization time-of-flight mass spectrometry (MALDI-TOF MS) (Voyager DE-ProR, PerSeptive Biosystems, Framingham, Massachusetts, USA). The chemical characteristics of PPE are the following: TPC of 83.00 ± 0.52 mg GAE g^−1^; major constituents are α-punicalagin, β-punicalagin, pedunculagin, and ellagic acid.

To make the extract compatible with the microbiological turbidimetric assays and facilitate long-term storage, the extract was concentrated using a rotary evaporator (Heidolph 36001270 Hei-Vap Precision Rotary Evaporator, Heidolph Instruments GmbH & Co. KG, Schwabach, Bavaria, Germany) and then lyophilized (Lio 5P Lyophilizer, Pascal Srl, Milan, Lombardy, Italy). The concentration of the lyophilized extracts in fresh solvent was subsequently adjusted according to the specific requirements of the performed assays.

### 2.2 Clinical isolates and growth conditions

The antimycotic activity of PPE was evaluated against three clinically significant fungal urogenital pathogens, *C. albicans* UGPCA25, *C. glabrata* UGPCG25 and *C. parapsilosis* UGPCP25. The tested clinical isolates were kindly provided by the Microbiology Laboratory of the University Hospital “Federico II” in Naples, Italy. They were obtained from vaginal mucosal samples and identified using the biochemical phenotyping method of the BD Phoenix™ Automated Microbiology System (Becton Dickinson, Franklin Lakes, New Jersey, United States), according to the manufacturer's instructions.

The fungal isolates were cultured under aerobic conditions at a controlled temperature of 37 ± 2 °C on the non-selective medium Potato Dextrose (PD) agar/broth (CONDA, Madrid, Spain), as well as on the selective chromogenic medium Candida Chromogenic Agar (CONDA, Madrid, Spain). A susceptibility profile for standard azoles—including climbazole, fluconazole, itraconazole, posaconazole, tioconazole, and voriconazole (Sigma-Aldrich, St. Louis, MO, USA)—was evaluated against the clinical isolates according to the Clinical and Laboratory Standards Institute (CLSI) guidelines (M27) (CLSI, 2022). These antifungals were selected as they represent agents commonly employed in both clinical and topical infections, providing a meaningful comparative assessment of the isolates' resistance patterns. The complete susceptibility data are reported in the Supplementary material (Supplementary Figure S1 and Supplementary Table S1). Among the tested agents, tioconazole was selected as the positive control in antimicrobial assays, as it consistently displayed the strongest inhibitory activity, with all three isolates showing the highest susceptibility to this compound. This choice ensured a reliable reference point for evaluating the relative efficacy of other treatments.

### 2.3 Antifungal assays

#### 2.3.1 Agar diffusion method

To evaluate the *in vitro* antimycotic activity of PPE against the tested *Candida* spp. isolates, an antimicrobial activity test was performed using the agar diffusion method, as described by Bauer et al. (1966), with some modifications, in accordance with the new guidelines from the Clinical and Laboratory Standards Institute (CLSI) [(Clinical and Laboratory Standards Institute (CLSI), 2017)]. Briefly, fungal strains were cultured in PD broth, and 100 μL aliquots of the microbial suspension, with an optical density (OD) of 0.5 at 600 nm, were spread onto PD agar. Subsequently, filter paper discs (6 mm in diameter, Oxoid, S.p.A., Rodano, Milan, Italy), impregnated with the extract (1 mg/disc), were placed onto the solid media. Tioconazole (1 mg/disc) was used as positive control, while the hydroethanolic extraction solution was used as negative control. The plates were incubated under aerobic conditions at 37 ± 2 °C for 24–48 h. After growth, the diameter (measured in mm) of the inhibition zones was determined, and antimycotic activities were expressed as the mean diameter of the inhibition zones (MDIZ) produced by the antifungal agents against the tested yeasts.

#### 2.3.2 Broth microdilution method

The susceptibility of yeast isolates to different concentrations of PPE (0, 2.5, 5, 10, 20, 30, 40 μg μL^−1^) was determined using the broth microdilution method with a standardized inoculum of 1 × 10^5^ CFU mL^−1^ (Colony-Forming Units/mL), in accordance with the new guidelines from the Clinical and Laboratory Standards Institute (CLSI) (Clinical and Laboratory Standards Institute (CLSI), 2017). Tioconazole (0, 0.05, 0.1, 0.2, 0.3, 0.4, 0.5, 0.6, 0.7, 0.8, 1.0, 2.0, 4.0 μg μL^−1^) was used as positive control, while the hydroethanolic extraction solution was used as negative control. The assay was performed in 96-well flat-bottom microplates (Nunc™ MicroWell, Thermo Scientific, Roskilde, Denmark). The minimum inhibitory concentration (MIC) was defined as the lowest concentration of antimycotic agent that prevented fungal growth. The minimum fungicidal concentration (MFC) was defined as the lowest concentration of antimycotic agent capable of killing 99% of the microorganisms present in the initial inoculum.

#### 2.3.3 Fungal fitness assay

To assess the inhibitory effect of PPE on the fitness of *C. albicans* UGPCA25, *C. glabrata* UGPCG25, and *C. parapsilosis* UGPCP25, the antimytotic activity of the tested extract over time was evaluated. Specifically, a fungal survival assay was conducted with increasing concentrations of the extract ranging from 5 μg μL^−1^ to 40 μg μL^−1^. To assess the survival of each fungal isolate, during the 144-h observation period, aliquots of serial dilutions of the microbial suspensions were spread onto PD agar, and the plates were incubated under aerobic conditions at 37 ± 2 °C for 24–48 h. After incubation, fungal colony counts were performed to determine the number of viable colonies.

#### 2.3.4 Membrane permeability assay

To evaluate the disruption of cell membrane integrity induced by PPE on the tested vaginal yeasts, a membrane permeability assay was performed to quantify the leakage of double-stranded DNA (dsDNA) and protein, according to Di Rosario et al. (2025), with minor modifications. Specifically, increasing concentrations of PPE corresponding to the MIC, 2 × MIC, and 4 × MIC were incubated with the microbial suspension (standard inoculum of 1 × 10^6^ cells mL^−1^) at 37 ± 2 °C with shaking at 200 rpm for 24 h. Triton X-100 (1%) and PD broth were used as positive and negative controls, respectively. At regular time intervals (0, 1, 2, and 4 h), samples were centrifuged at 3,000 rpm for 10 min (Centrifuge 5804 R, Eppendorf, Milan, Italy), and the cell-free supernatants were filtered through 0.45 μm syringe filters (Merck-Millipore, Darmstadt, Germany). The filtrates were then analyzed using a UV-Visible NanoDrop™ Spectrophotometer (Thermo Fisher Scientific, Waltham, MA, USA) at 260 and 280 nm to quantitatively determine dsDNA and protein leakage, respectively. The amount of released dsDNA was expressed in ng μL^−1^, while released protein was expressed in mg mL^−1^. Three replicates were performed for each experiment using three independent cultures.

### 2.4 Antigerminative assays

#### 2.4.1 Serum-induced germ tube inhibition assay

The ability of PPE to inhibit germ tube formation by *Candida* spp. clinical isolates was assessed using the serum-induced germ tube inhibition assay, following the method of Sadowska et al. (2014) with minor modifications. Fungal inocula (1 × 10^6^ cells mL^−1^) were prepared and incubated at 37 ± 2 °C in RPMI 1640 medium (Sigma-Aldrich, St. Louis, Missouri, USA), supplemented with 10% (v/v) fetal bovine serum (FBS, Euroclone S.p.A., Milan, Italy) in the absence (control) or presence of increasing concentrations of PPE (5, 10, 20, and 40 μg μL^−1^). At 0, 2, 4, 8, 24, and 48 h, cellular morphology was assessed for each culture using a Burker counting chamber and a trinocular optical microscope (Motic B1 series, model B1-223, 40X/0.65 objective, WD 0.17 mm). Germ tube-forming cells (GTFs) were counted. Cells were considered positive for germination if the germ tube was at least twice the length of the yeast cell. Results were expressed as mean GTF/mL of inoculum ± standard deviation (SD). The percentage of GTFs (GTF%) after treatment with pomegranate extracts was calculated relative to the untreated control (considered as 100%). Additionally, the minimum germ tube inhibition concentration (MGIC) was determined as the lowest concentration of the antifungal agent causing ≥90% inhibition of germ tube formation after 24 h, providing a standardized measure of antigemination activity. Tioconazole was used as a positive control.

#### 2.4.2 Germ tubes analysis by fluorescence microscopy

At specific incubation time points, fungal germ tubes treated with PPE (40 μg μL^−1^) were also analyzed by fluorescence microscopy through staining with fluorescein isothiocyanate-conjugated Concanavalin A (FITC-ConA, C7642, Sigma-Aldrich, St. Louis, Missouri, USA), which binds mannose and glucose residues on both the *Candida* yeast cells and the germ tubes. Specifically, the fungal cultures were stained with FITC-ConA at a concentration of 0.2 mg mL^−1^ and incubated at room temperature for 15 min with gentle shaking at 150 rpm, in the dark to prevent photobleaching. After incubation, 20 μL aliquots were placed onto clean glass microscope slides and observed under a fluorescence microscope (Nikon ECLIPSE Ti, with digital camera DS-Qi2, Nikon Instruments Inc., Tokyo, Japan) equipped with FITC filters (excitation at 490 nm and emission at 520 nm). Images were captured using a 40 × objective lens (0.65 NA, S, WD 0.53 mm), enabling clear visualization of PPE's inhibitory effect on germ tube formation. Image acquisition and analysis were performed using NIS-Elements C software (Nikon Instruments Inc., Tokyo, Japan).

### 2.5 Antibiofilm assays

#### 2.5.1 Tissue culture plate method

To evaluate the ability of PPE to inhibit biofilm formation by *C. albicans* UGPCA25, *C. glabrata* UGPCG25, and *C. parapsilosis* UGPCP25, the Tissue Culture Plate Method (TCPM) was performed as described by Sateriale et al. (2020b), with minor modifications. Specifically, increasing concentrations of PPE (MIC, 2 × MIC, 3 × MIC, and 4 × MIC) were dispensed into the wells of a 96-well flat-bottom microplate (Nunc™ MicroWell 96-well plates, Thermo Scientific, Roskilde, Denmark). Sterile PD broth was added to the wells designated as negative controls. Tioconazole was used as a positive control. Subsequently, 0.1 mL of each fungal culture, adjusted to an OD600 of 0.5, was added to each well, reaching a final volume of 0.2 mL. Wells containing only 0.2 mL of PD broth were used as blanks. The plates were incubated at 37 ± 2 °C for 48 h without shaking to allow yeast adhesion to the well surfaces. After incubation, non-adherent and planktonic cells were removed by washing the wells three times with phosphate-buffered saline (1 × PBS, pH 7.3, Sigma-Aldrich, Merck KGaA, Darmstadt, Germany). The adherent biofilms were then fixed with 85% ethanol (Sigma-Aldrich, Merck KGaA, Darmstadt, Germany) for 15 min and stained with 0.2% crystal violet (Sigma-Aldrich, Merck KGaA, Darmstadt, Germany) for 5 min. Excess stain was removed by rinsing with deionized water, and the plates were dried upside down in an incubator at 30 °C for 10 min.

Following drying, 85% ethanol was added to the stained wells to solubilize the crystal violet, and the absorbance (OD) was measured at 600 nm using a microplate reader (Bio-Rad Model 680 Microplate Reader, Bio-Rad Laboratories, Hercules, California, USA). The measurement of the optical density of each fungal culture (OD_fc_), and the comparison with values measured for sterile Potato Dextrose broth used as the negative control (OD_nc_), allowed to classify the fungal cultures as non-adherent (OD_fc_ ≤ O_nc_), weakly adherent (OD_nc_ < OD_fc_ ≤ 2 OD_nc_), moderately adherent (2 OD_nc_ < OD_fc_ ≤ 4 OD_nc_) and strongly adherent (4 OD_nc_ < OD_fc_). Minimum Biofilm Inhibition Concentration (MBIC) was defined as the lowest concentration of antifungal agent able to produce fungal biofilm inhibition.

#### 2.5.2 Eradication assay

To evaluate the ability of PPE to eradicate mature biofilms formed by the tested fungal isolates, an adapted version of the tissue culture plate method (TCPM) was used. Specifically, 0.2 mL aliquots of each fungal culture (adjusted to an OD_600nm_ of 0.5) were dispensed into the wells of flat-bottomed 96-well microplates. Wells containing 0.2 mL of sterile PD broth without fungal inoculum served as negative controls. The plates were incubated under aerobic conditions at 37 ± 2 °C for 48 h without shaking to allow biofilm formation and cell adhesion to the well surfaces. Following incubation, the medium was gently removed from each well and increasing concentrations of PPE equal to or greater than the previously determined MBIC (MBIC, 2 × MBIC, and 3 × MBIC) were added. In the negative control wells, the same volume of PD broth was used in place of the extract. In parallel, tioconazole was employed as a positive control. Additional aliquots of PD broth were added to each well to reach a final volume of 0.2 mL. The plates were then incubated again at 37 ± 2 °C for 24 h under aerobic conditions. After this second incubation, non-adherent cells and residual extract were removed by washing the wells with PBS, followed by fixation with 85% ethanol, staining with 0.2% crystal violet for 5 min, and a final wash with deionized water to remove excess dye. The absorbance was measured at 600 nm using a microplate reader. The results were expressed as a biofilm eradication index (BEI%) according to the following formula, adapted from Bakkiyaraj et al. (2013):


$$\begin{array}{l} BEI\%={\left(\frac{{OD_{control}-\;OD}_{test}}{OD_{control}}\right)}^{*}100 \end{array}$$

where OD_control_ is the average optical density of untreated mature fungal biofilms, and OD_test_ is the average optical density of mature biofilms treated with PPE. The lowest concentration of antifungal agent capable of completely disrupting the preformed biofilms was defined as the Minimum Biofilm Eradication Concentration (MBEC).

#### 2.5.3 Biofilm architecture analysis by scanning electron microscopy

To examine the structural morphology and integrity of biofilms formed by *Candida* isolates, Scanning Electron Microscopy (SEM) analyses were performed, assessing fungal biofilms grown on polyvinyl chloride (PVC), a material commonly found in urinary catheters and other medical devices, where *Candida* biofilms are known to develop, particularly in nosocomial settings. In brief, circular PVC discs (1 cm in diameter) were placed in the wells of sterile 24-well microplates and inoculated with fungal suspensions in PD broth (OD600 of 0.5) in the absence of PPE at MBIC vlaues, to allow for biofilm formation. The plates were incubated under aerobic conditions at 37 ± 2 °C for 48 h. After incubation, the medium was carefully removed and the PVC discs were gently washed three times with PBS to eliminate non-adherent cells. The remaining adherent biofilms were fixed with 4% paraformaldehyde (Sigma-Aldrich, Merck KGaA, Darmstadt, Germany) in PBS for 1 h at room temperature and dehydrated through a graded ethanol series (30, 50, 70, 90, and 100%). Samples were then mounted on aluminum stubs using double-sided carbon adhesive tape and subsequently coated with a thin layer of gold using a Q150R ES Sputter Coater (Quorum Technologies, Lewes, UK). Imaging was performed using a Scanning Electron Microscope (SEM; Zeiss EVO 15 HD VPSEM) operating at an accelerating voltage of 15 kV. Micrographs were acquired at magnifications of 500 × , 2,000×, and 5,000×, allowing for high-resolution visualization of biofilm architecture and surface modifications induced by PPE treatment.

## 3 Results

### 3.1 *In vitro* antimycotic activity of PPE

Preliminary screening of the *in vitro* antimycotic activity of the hydroethanolic PPE revealed that the extract effectively inhibits the growth of clinical isolates of *C. albicans* UGPCA25, *C. glabrata* UGPCG25, and *C. parapsilosis* UGPCP25, as evidenced by inhibition zones observed through the agar diffusion assay. The mean diameters of the inhibition zones (MDIZ) against tested urogenital pathogenic isolates are reported in Table 1. The inhibition zones (mm) are expressed as the mean of triplicate assays ± standard deviation. The MDIZs at 1 mg/disc were 14.67 ± 2.05 mm for *C. albicans* UGPCA25, 25.33 ± 3.09 mm for *C. glabrata* UGPCG25, and 23.00 ± 0.82 mm for *C. parapsilosis* UGPCP25. Tioconazole was tested as positive control, showing antimycotic efficacy against all tested strains, while no effects were observed for the hydroethanolic extraction solution used as negative control (data not shown).

**Table 1:** *In vitro* antimycotic activity of hydroethanolic PPE and tioconazole against *Candida* spp. isolates by the agar diffusion method.

**Antifungal agent**	**MDIZ (mm)**		
	***Candida albicans*** **UGPCA25**	***Candida glabrata*** **UGPCG25**	***Candida parapsilosis*** **UGPCP25**
PPE (1 mg/disc)	14.67 ± 2.05 ^***^	25.33 ± 3.09 ^**^	23.00 ± 0.82 ^*^
TCZ (1 mg/disc)	23.00 ± 7.26	27.67 ± 1.25	26.67 ± 3.77

The inhibition zones (mm) are expressed as the mean of triplicate assays ± standard deviation. Statistical significance was assessed using Student's *t*-test to compare the activity of PPE and tioconazole for each *Candida* isolate; asterisks indicate significance (^*^p < 0.05; ^**^p < 0.01; ^***^p < 0.001). MDIZ, mean diameter of inhibition zones; PPE, pomegranate peel extract; TZC, tioconazole.

The antimycotic activity of PPE against *Candida* vaginal isolates was also quantitatively evaluated by the microdilution method, according to the CLSI guidelines (CLSI, 2022). For *C. albicans* UGPCA25, the MIC value of PPE was 20 μg μL^−1^, and the MFC value was 40 μg/μL (Table 2). The PPE exhibited a MIC value of 10 μg μL^−1^ for *C. glabrata* UGPCG25, while the MBC value was 20 μg μL^−1^ (Table 2). The PPE exerted a strong fungistatic and fungicidal effect also against *C. parapsilosis* UGPCP25 with MIC and MFC values of 10 μg μL^−1^ and 20 μg μL^−1^, respectively (Table 2). All fungal isolates tested in this study were sensitive to tioconazole tested as a positive control.

**Table 2:** Antimycotic activity of hydroethanolic PPE and tioconazole against clinical isolates of *Candida* spp.

**Antifungal agent**	***Candida albicans*** **UGPCA25**		***Candida glabrata*** **UGPCG25**		***Candida parapsilosis*** **UGPCP25**	
	**MIC (**μ**g** μ**L**^−1^**)**	**MFC (**μ**g** μ**L**^−1^**)**	**MIC (**μ**g** μ**L**^−1^**)**	**MFC (**μ**g** μ**L**^−1^**)**	**MIC (**μ**g** μ**L**^−1^**)**	**MFC (**μ**g** μ**L**^−1^**)**
PPE	20^****^	40^****^	10^****^	20^****^	10^****^	20^****^
TCZ	0.2	0.4	0.1	0.2	0.1	0.2

Statistical significance was assessed using Student's *t*-test to compare the activity of PPE and tioconazole for each *Candida* isolate; asterisks indicate significance (^****^p < 0.0001). PPE, pomegranate peel extract; TZC, tioconazole; MIC, minimum inhibitory concentration; MFC, minimum fungicidal concentration.

### 3.2 Effects of PPE on fungal survival

To verify the effect of PPE on the fitness of selected pathogens, the survival rate of each fungal isolate was evaluated for 144 h, with increasing concentrations of hydroethanolic PPE. Evaluation of viable counts showed a dose-dependent fungistatic and fungicidal activity of PPE against *Candida* isolates.

**Figure 1:**
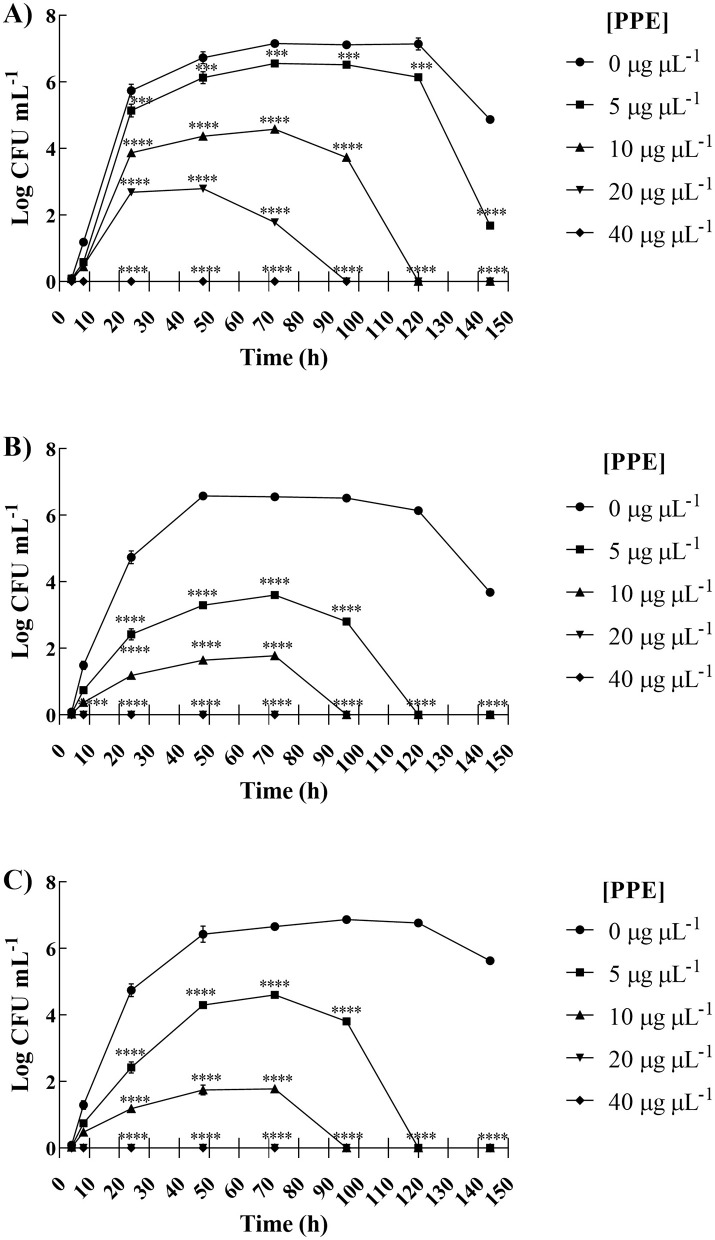
Inhibitory effect of hydroethanolic pomegranate peel extract (PPE) on fitness of clinical *Candida* spp. isolates. Survival of *C. albicans* UGPCA25 **(A)**, *C. glabrata* UGPCG25 **(B)**, and *C. parapsilosis* UGPCP25 **(C)** was assessed in the absence (control) and presence of PPE. Results are expressed as Log colony-forming units (CFU) per mL of yeast inoculum and are presented as mean ± standard deviation. Asterisks (***p < 0.001; ****p < 0.0001) indicate statistical significance assessed by two-way ANOVA with Bonferroni correction, comparing treated cultures to untreated fungal controls.

As shown in Figure 1A, a fungistatic effect was observed for *C. albicans* UGPCA25 isolate when treated with 5 μg μL^−1^ of PPE. The exposure to 10 and 20 μg μL^−1^ of PPE caused a significant reduction of fungal survival of *C. albicans* isolate in the first 24 h, achieving fungal cell death at the end of the observation time (Figure 1A). Moreover, with a concentration of 40 μg μL^−1^, a fungicidal effect was observed already after 8 h of exposure. For *C. glabrata* UGPCG25 isolate, the assay showed a reduction in fungal viability over time even at concentrations of PPE corresponding to 5 and 10 μg μL^−1^, up to obtaining a fungicidal action upon exposure to 20 and 40 μg μL^−1^ (Figure 1B). Similarly, the same trend has been recorded for *C. parapsilosis* UGPCP25 isolate (Figure 1C). Overall, these findings clearly indicate that the hydroethanolic PPE is able to effectively *in vitro* antagonize the growth of fungal urogenital pathogens.

### 3.3 Evaluation of the effect of PPE on yeast cell membrane integrity

**Figure 2:**
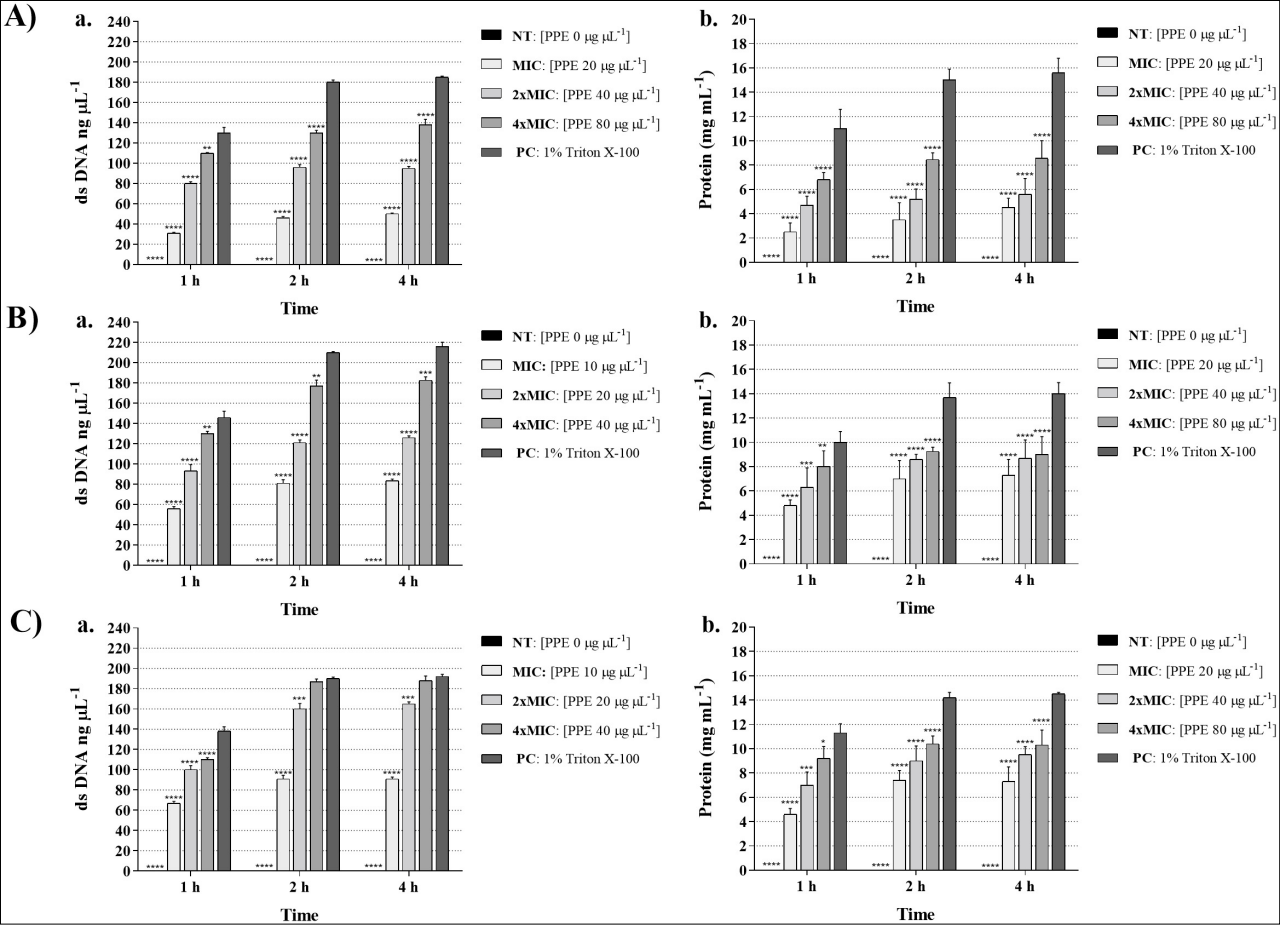
Effect of hydroethanolic pomegranate peel extract (PPE) on yeast cell membrane integrity. dsDNA (ng/μL) and protein (mg/mL) leakage induced by increasing concentrations of PPE (MIC, 2 × MIC, 4 × MIC) against *C. albicans* UGPCA25 **(A)**, *C. glabrata* UGPCG25 **(B)**, and *C. parapsilosis* UGPCP25 **(C)**. NT indicates untreated control, and 1% Triton X-100 was used as positive control (PC). Results are presented as mean ± standard deviation. Asterisks (**p < 0.01; ***p < 0.001; ****p < 0.0001) indicate statistical significance assessed by two-way ANOVA with Bonferroni correction, comparing treated samples to untreated controls.

The disruption of yeast membrane integrity induced by PPE was demonstrated by the leakage of genetic and protein material through the cell membrane. The alteration of *Candida* cell membrane integrity was evaluated by measuring the release of cellular components, based on the absorbance at 260 and 280 nm in the supernatants of yeast cultures treated with increasing concentrations of PPE. The results demonstrated that the release of both dsDNA and protein content increased in a dose-dependent manner in yeasts exposed to PPE at MIC, 2 × MIC, and 4 × MIC (Figure 2). Notably, a greater effect was observed against *C. glabrata* and *C. parapsilosis*, with dsDNA release values ranging from 93.31 ± 6.12 ng μL^−1^ (1 h treatment with PPE at MIC against *C. glabrata*, Figure 2B-a) to 188.00 ± 5.50 ng μL^−1^ (4 h treatment with PPE at 4 × MIC against *C. parapsilosis*, Figure 2C-a). Similarly, protein release increased up to 9.0 ± 1.47 mg/mL and 10.30 ± 1.23 mg mL^−1^ for *C. glabrata* and *C. parapsilosis*, respectively, after treatment of 4 h with the highest PPE concentration tested (Figures 2B-b, C-b). Likewise, in *C. albicans*, dsDNA release ranged between 31.00 ± 0.74 and 138.00 ± 5.76 ng μL^−1^ (Figure 2A-a), while protein leakage varied from 2.50 ± 0.74 to 8.56 ± 1.46 mg mL^−1^ (Figure 2A-b) in response to increasing PPE concentrations throughout the observation period, indicating the greater resistance of the tested urogenital *C. albicans* isolate. A 1% Triton X-100 solution was used as a positive control and confirmed the release of dsDNA and protein through the yeast membrane (Figure 2).

### 3.4 *In vitro* antigerminative activity of PPE

**Figure 3:**
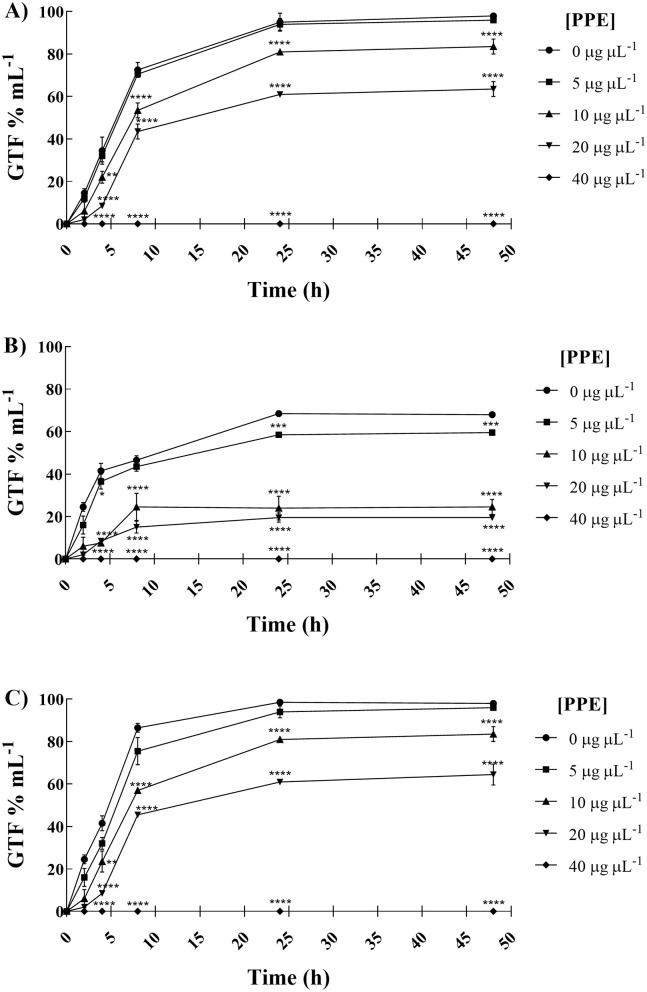
Anti-germinative effect of hydroethanolic pomegranate peel extract (PPE) against *C. albicans* UGPCA25 **(A)**, *C. glabrata* UGPCG25 **(B)**, and *C. parapsilosis* UGPCP25 **(C)** isolates. The figure shows the percentage of germ tube-forming cells (GTF%) in the absence and presence of PPE at increasing concentrations. Results are presented as mean ± standard deviation. Asterisks (**p < 0.01; ***p < 0.001; ****p < 0.0001) indicate statistical significance assessed by two-way ANOVA with Bonferroni correction, comparing treated cultures to untreated fungal controls.

**Figure 4:**
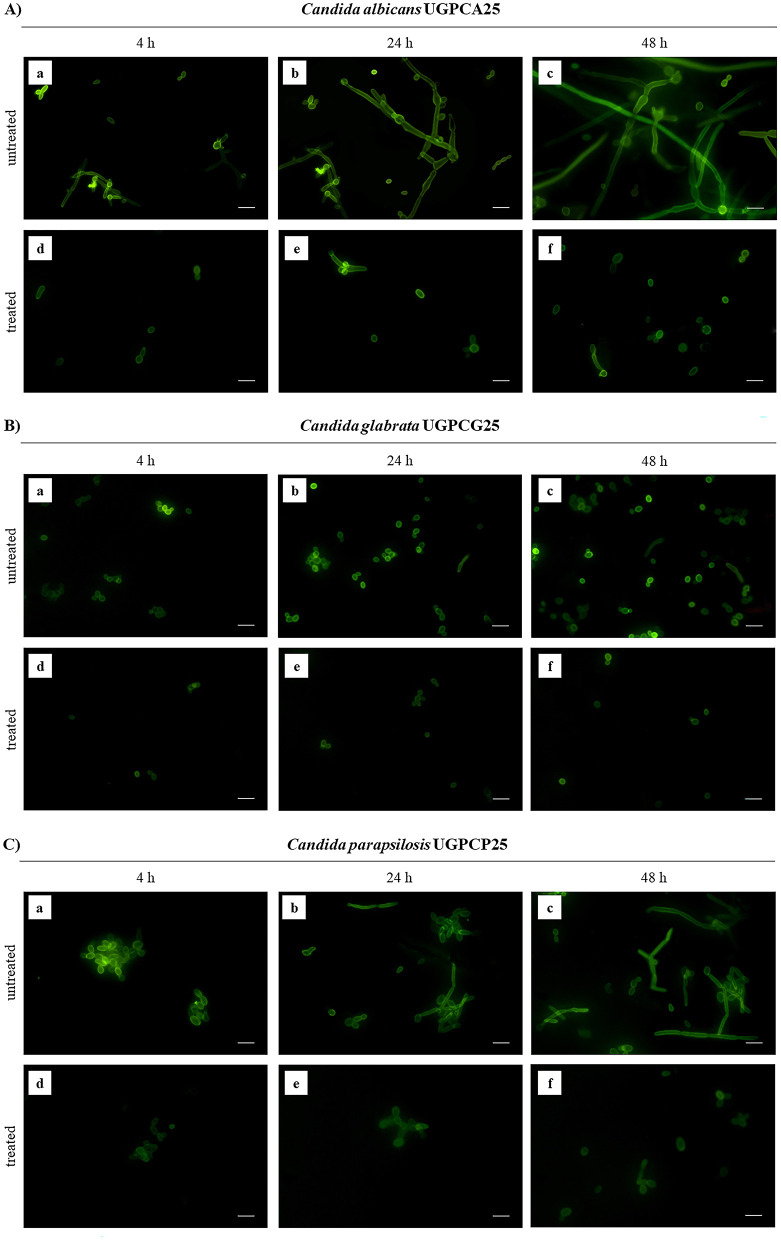
Germ tube formation by *Candida* isolates untreated and treated with hydroethanolic pomegranate peel extract (PPE) at 4, 24, and 48 h. **(A)** *C. albicans* UGPCA25 untreated **(a–c)** and PPE-treated **(d–f)**; **(B)** *C. glabrata* UGPCG25 untreated **(a–c)** and PPE-treated **(d–f)**; **(C)** *C. parapsilosis* UGPCP25 untreated **(a–c)** and PPE-treated **(d–f)**. Image acquisition *via* Nikon ECLIPSE Ti fluorescence microscope (40X objective, 0.65 NA, S, WD 0.53 mm) and NIS-Elements C software. Scale bar: 10 μm.

The polyphenolic PPE was tested to assess its antigerminative activity against the three selected clinical fungal isolates. The effect of increasing concentrations (5, 10, 20, and 40 μg μL^−1^) of PPE on germ tube formation and hyphal growth of *C. albicans* UGPCA25, *C. glabrata* UGPCG25, and *C. parapsilosis* UGPCP25 is shown in Figure 3. The extract demonstrated the ability to inhibit germ tube formation of *Candida albicans* within the first 4 h of incubation at a concentration of 20 μg μL^−1^. At this concentration, the percentage reduction in germinative cells was 22.00, 41.50, 52.00, and 51.50% after 4, 8, 24, and 48 h of incubation, respectively (Figure 3A). These values were calculated with respect to the negative control, where, in the absence of extract, nearly 100% of cells exhibited germ tube formation after 48 h. Similar inhibitory effects were observed for the *C. parapsilosis* isolate (Figure 3C). In contrast, the *C. glabrata* isolate was characterized by a low propensity for germ tube formation and PPE showed a significant antigerminative activity at lower concentrations vs. this isolate, with a 61.50% reduction observed at 10 μg μL^−1^ after just 8 h of incubation (Figure 3B). The minimum germ tube inhibition concentration (MGIC) of PPE was 40 μg μL^−1^ for all tested isolates, providing a standardized measure of its antigemination activity. Tioconazole was used as a positive control, exhibiting MGIC values of 1.0 μg μL^−1^ for *C. albicans* and 0.5 μg μL^−1^ for *C. glabrata* and *C. parapsilosis*. The antigerminative effect of PPE at 40 μg μL^−1^ was further confirmed by fluorescence microscopy (Figure 4), showing a marked reduction or complete absence of germ tube formation across all incubation times.

### 3.5 *In vitro* antibiofilm activity of PPE

**Figure 5:**
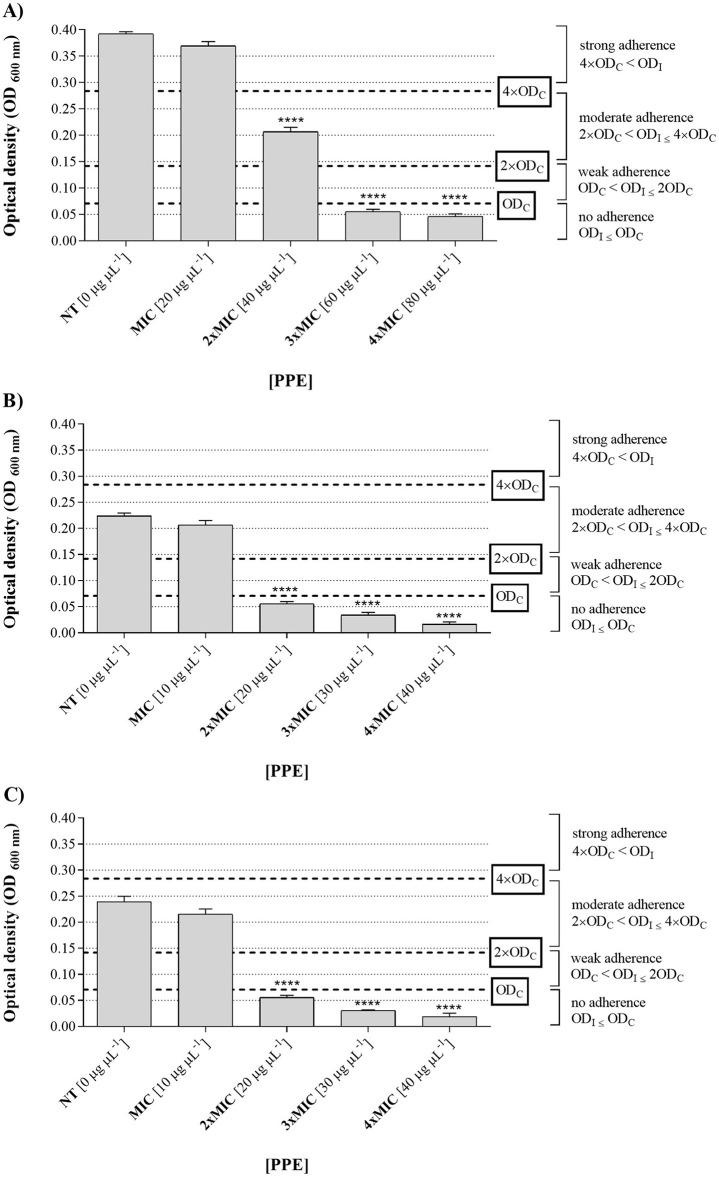
Antibiofilm activity of hydroethanolic pomegranate peel extract (PPE) against *Candida* isolates. Adherence levels of biofilms formed by *C. albicans* UGPCA25 **(A)**, *C. glabrata* UGPCG25 **(B)**, and *C. parapsilosis* UGPCP25 **(C)** in the absence (NT) and presence of increasing concentrations of PPE (MIC, 2 × MIC, 3 × MIC, and 4 × MIC). Results are presented as mean ± standard deviation. Asterisks (**** *p* < 0.0001) indicate statistical significance assessed by two-way ANOVA with Dunnett's correction, comparing treated cultures to untreated fungal controls. NT, not treated; MIC, minimum inhibitory concentration.

The *in vitro* assays revealed that PPE exhibited strong antibiofilm activity, significantly inhibiting the biomass of the tested fungal isolates compared to the untreated control, as shown in Figure 5. Based on the optical density measurements of biofilms grown in the presence of increasing concentrations of the extract (0, 20, 40, 60, and 80 μg μL^−1^ for *C. albicans* UGPCA25; 0, 10, 20, 30, and 40 μg μL^−1^ for *C. glabrata* UGPCG25 and *C. parapsilosis* UGPCP25), and the comparison with the optical density of the sterile PD broth medium used as the negative control, the *Candida* spp. clinical isolates were classified as non-adherent, weakly adherent, moderately adherent, and strongly adherent.

In particular, in the absence of extract (0 μg μL^−1^) and at a concentration of 20 μg μL^−1^, the *C. albicans* isolate was strongly adherent (4 OD_nc_ < OD_fc_), while increasing concentrations of the tested extract led to a gradual decrease in adherence (Figure 5A). Notably, at a concentration of 40 μg μL^−1^, the isolate showed moderate adherence (2 OD_nc_ < OD_fc_ ≤ 4 OD_nc_), whereas at concentrations 3 and 4 times the MIC, i.e., 60 and 80 μg μL^−1^, the microbial isolate showed no adherence (OD_fc_ ≤ O_nc_) (Figure 4A). Based on these results, the concentration of 60 μg μL^−1^ was identified as the MBIC for PPE against the tested *C. albicans* vaginal isolate (Table 3). Regarding the fungal isolates of *C. glabrata* and *C. parapsilosis*, both moderately adherent isolates, they showed a loss of adherence at a lower concentration of PEE, specifically at 20 μg μL^−1^ (Figures 5B, C), which was identified as the MBIC for both fungal isolates (Table 3).

**Table 3:** Antibiofilm activity of hydroethanolic PPE and tioconazole against clinical isolates of *Candida* spp.

**Antifungal agent**	***Candida albicans*** **UGPCA25**		***Candida glabrata*** **UGPCG25**		***Candida parapsilosis*** **UGPCP25**	
	**MBIC (**μ**g** μ**L**^−1^**)**	**MBEC (**μ**g** μ**L**^−1^**)**	**MBIC (**μ**g** μ**L**^−1^**)**	**MBEC (**μ**g** μ**L**^−1^**)**	**MBIC (**μ**g** μ**L**^−1^**)**	**MBEC (**μ**g** μ**L**^−1^**)**
PPE	60^****^	120^****^	20^****^	40^****^	20^****^	60^****^
TCZ	1.0	2.0	0.6	1.0	0.8	1.0

Statistical significance was assessed using Student's *t*-test to compare the activity of PPE and tioconazole for each *Candida* isolate; asterisks indicate significance (^****^p < 0.0001). PPE, pomegranate peel extract; TZC, tioconazole; MBIC, minimum biofilm inhibitory concentration; MBEC, minimum biofilm eradication concentration.

**Figure 6:**
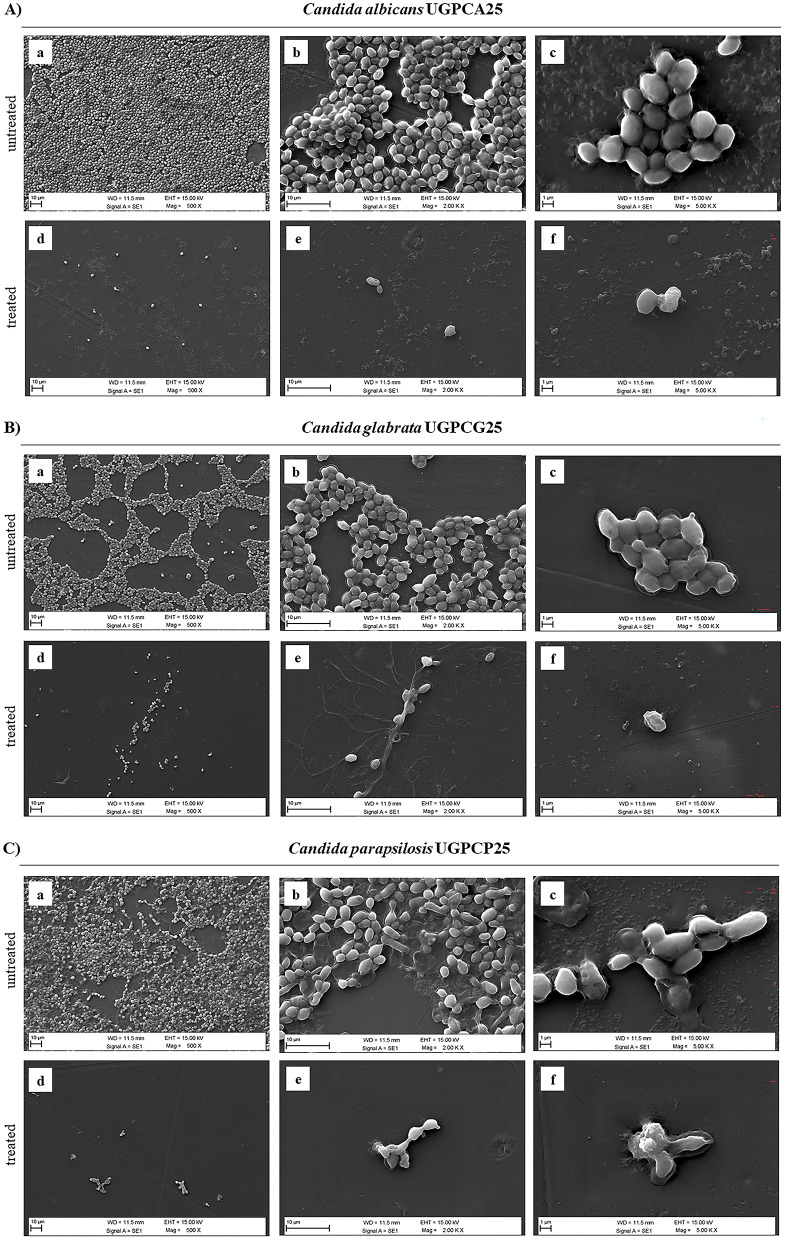
Scanning Electron Microscopy (SEM) images of biofilms formed by *Candida* isolates, untreated and treated with hydroethanolic pomegranate peel extract (PPE). **(A)** *C. albicans* UGPCA25 untreated **(a–c)** and PPE-treated **(d–f)**; **(B)** *C. glabrata* UGPCG25 untreated **(a–c)** and PPE-treated **(d–f)**; **(C)** *C. parapsilosis* UGPCP25 untreated **(a–c)** and PPE-treated **(d–f)**. Image acquisition *via* Zeiss EVO 15 HD VPSEM scanning electron microscopy at three magnifications (500×, 2,000×, and 5,000×).

To further investigate the effects of PPE on biofilm structure, SEM imaging was performed to assess the biofilm architecture of the tested *Candida* isolates (*C. albicans* UGPCA25, *C. glabrata* UGPCG25, and *C. parapsilosis* UGPCP25) grown on polyvinyl chloride (PVC) (Figure 6). At the MBIC concentrations, SEM analyses revealed a marked reduction in both *Candida* cells and biofilm biomass in the PPE-treated samples compared to the untreated controls. In the control groups, *Candida* cells exhibited strong adhesion to the PVC surface, forming dense and well-organized biofilms (Figures 6A-a, B-a, C-a). At 2,000 × magnification, the extracellular matrix enveloping the cells was clearly evident, firmly anchoring them to the PVC substrate (Figures 6A-b, B-b, C-b), while images at 5,000 × magnification revealed intact cell surfaces and a cohesive biofilm matrix (Figures 6A-c, B-c, C-c). In contrast, PPE-treated samples showed a pronounced disruption of biofilm architecture. A significant reduction in cell adhesion and biomass loss were consistently observed across all tested *Candida* isolates (Figures 6A-d, B-d, C-d). SEM images at 2,000 × (Figures 6A-e, B-e, C-e) and 5,000 × magnifications (Figures 6A-f, 6B-f, and 6C-f) revealed substantial degradation of the exopolysaccharide matrix, accompanied by evident damage to cellular integrity. These findings suggest that PPE markedly compromises *Candida* spp. biofilm integrity, thereby inhibiting biofilm formation and stability.

**Figure 7:**
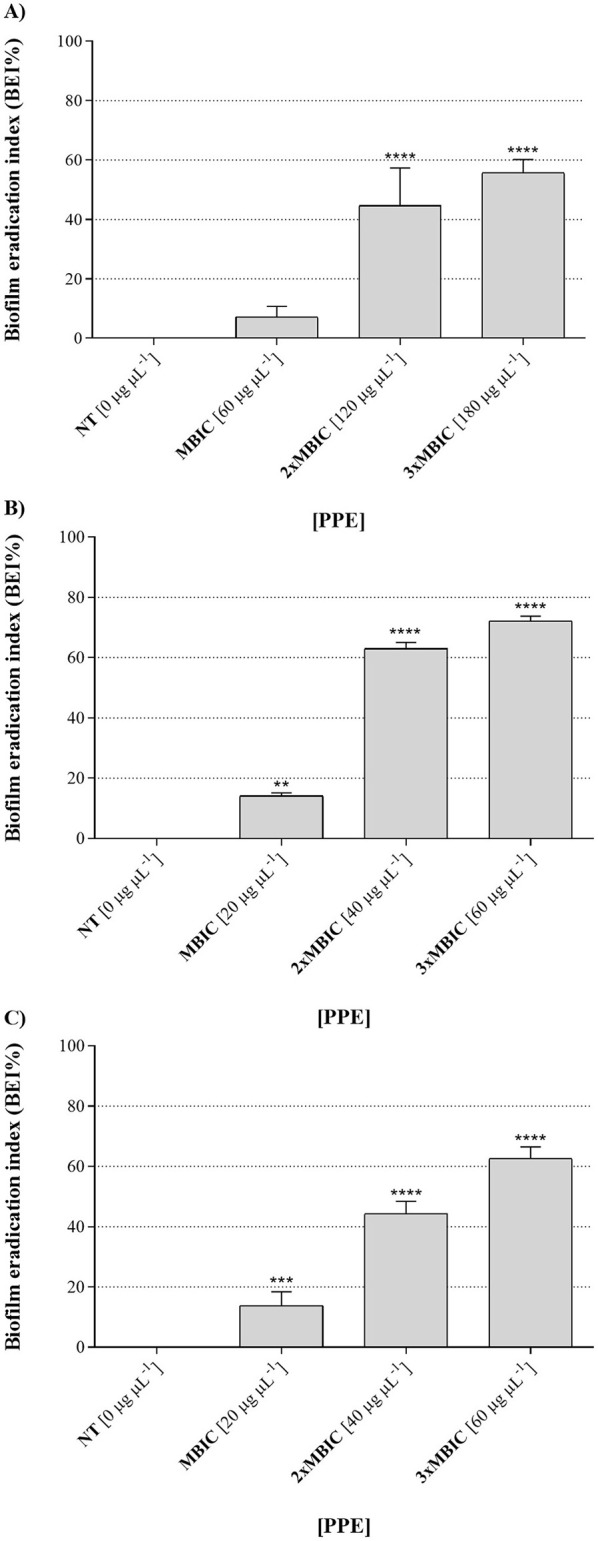
Biofilm eradication activity of hydroethanolic pomegranate peel extract (PPE) against *Candida* isolates. The figure shows the percentage of biofilm destruction for *C. albicans* UGPCA25 **(A)**, *C. glabrata* UGPCG25 **(B)**, and *C. parapsilosis* UGPCP25 **(C)** clinical isolates in the presence of MBIC and higher concentrations (2 × MBIC and 3 × MBIC) of PPE. Results are presented as mean ± standard deviation. Asterisks (** *p* < 0.01; *** *p* < 0.001; **** *p* < 0.0001) indicate statistical significance assessed by two-way ANOVA with Dunnett's correction, comparing treated cultures to untreated fungal controls. NT, not treated; MBIC, Minimum biofilm inhibitory concentration.

Furthermore, the hydroethanolic PPE proved effective not only in inhibiting biofilm formation by the tested fungal isolates, preventing fungal adhesion, but also in eradicating pre-formed biofilms. It was found that mature biofilms of all the fungal isolates were significantly altered at increasing concentrations of the polyphenolic extract (Figure 7), with biofilm eradication indices reaching approximately 60% for *C. albicans* UGPCA25 (Figure 7A), 73% for *C. glabrata* UGPCG25 (Figure 7B), and 62% for *C. parapsilosis* UGPCP25 (Figure 7C) at a concentration three times higher than the MBIC value. The MBEC values, assigned to the minimum concentration of each antimicrobial agent capable of destroying pre-formed biofilms, are shown in Table 3.

## 4 Discussion

Fungal infections, particularly those caused by resistant *Candida* species, pose a rising threat to global health, limiting treatment efficacy and escalating morbidity, mortality, and healthcare costs (Arendrup and Patterson, 2017; Mudenda, 2024). This growing challenge underscores the urgent need to develop new antifungal agents, such as those derived from natural sources, which may offer safer, more effective, and sustainable alternatives to current treatments. Among these, pomegranate (*Punica granatum* L.) has attracted considerable attention for its wide range of pharmacological properties, including antioxidant, anti-inflammatory, anticancer, and antimicrobial effects (Singh et al., 2023). Notably, pomegranate peel is rich in bioactive compounds, including polyphenols and other antioxidants, often present at higher concentrations than in the edible portions of the fruit (Pagliarulo et al., 2016). With the increasing production of pomegranate-based products, this by-product has emerged as an abundant and valuable source for the development of extracts rich in antimicrobial phytochemicals.

In this study, we investigated the *in vitro* antifungal activity of hydroethanolic pomegranate peel extract (PPE) against urogenital clinical isolates of *C. albicans, C. glabrata*, and *C. parapsilosis*, encompassing both classical and emerging etiological agents of urogenital infections. To provide a comparative analysis, the antifungal activity of PPE was evaluated alongside tioconazole, a topical azole commonly employed for the management of superficial *Candida* infections. Tioconazole was selected as a positive control due to its consistently high activity against the tested isolates, providing a reliable benchmark for evaluating the relative potency of PPE. This comparison offers insights into the potential of PPE as an alternative or complementary therapy, particularly against strains exhibiting variable susceptibility to other conventional azoles, with relevance for topical applications.

Preliminary screening of PPE antimicrobial activity using the agar diffusion method revealed inhibition zones ranging from approximately 14.6 mm to 25.3 mm (Table 1), indicating a notable antifungal effect across all tested isolates. These findings were corroborated by quantitative microdilution assays, which demonstrated MIC values between 10 and 20 μg μL^−1^ and MFC values up to 40 μg μL^−1^ (Table 2). These results underscore the fungistatic and fungicidal potential of pomegranate peel extract (PPE), especially against *C. glabrata* and *C. parapsilosis*, which exhibited greater sensitivity compared to *C. albicans*. The antifungal efficacy of PPE aligns with recent studies indicating that polyphenol-rich plant extracts, including pomegranate peel extracts, demonstrate significant activity against both susceptible and drug-resistant fungal pathogens (Khan et al., 2023; Ferreira et al., 2025). However, most recent studies on the anti-*Candida* effects of PPE have concentrated on oral isolates (Bassiri-Jahromi et al., 2018; Tavazo Zadeh et al., 2023; Siddique et al., 2024). In contrast, our research specifically examines azole-resistant and emerging vaginal isolates, pathogens that are of increasing clinical concern in vulvovaginal candidiasis and remain underexplored in current literature. Moreover, the majority of previously published studies have utilized alcoholic extracts with ethanol or methanol concentrations exceeding 80% (Bassiri-Jahromi et al., 2015; Siddique et al., 2024; Ferreira et al., 2025). In contrast, we employed 50% hydroethanolic extracts, providing a more sustainable, eco-friendly, and non-cytotoxic alternative, as demonstrated in our previous work. Notably, recent reports on pomegranate peel extracts against *Candida* spp. have indicated MIC and MFC values ranging from 5 to 50 mg mL^−1^ and 10 to 150 mg mL^−1^, respectively (de Oliveira et al., 2013; Hamrita et al., 2022; Tavazo Zadeh et al., 2023). Therefore, the PPE evaluated in this study demonstrates antifungal activity comparable to—and in some cases exceeding—previously reported values in the literature.

The fungal survival curves (Figure 1) revealed a clear time- and dose-dependent fungicidal activity of PPE against all three *Candida* species. For *C. albicans*, complete killing was achieved within 144 h at 10–20 μg μL^−1^ and already within 8 h at 40 μg μL^−1^; similarly, PPE exerted fungicidal activity against *C. glabrata* and *C. parapsilosis* by 144 h at 20 μg μL^−1^ and within 8 h at 40 μg μL^−1^. Importantly, a previous Authors' study demonstrated that these PPE concentrations are non-cytotoxic to human cell lines (Scaglione et al., 2024), underscoring PPE's potential as a safe and effective antifungal agent.

In our investigation of the antifungal mechanisms of PPE, we revealed that this extract exerts its activity through the disruption of yeast cell membrane integrity. The membrane of *Candida* species represents a dynamic and selectively permeable barrier that regulates the transport of ions, nutrients, and metabolites, thereby maintaining the homeostasis essential for cellular viability (Eliaš et al., 2024). Consequently, even minor perturbations in membrane permeability can compromise cell metabolism and ultimately lead to cell death. Evaluating the structural integrity of the fungal membrane, therefore, provides valuable insights into the mechanism of antifungal action. Several studies have established that the release of intracellular components, such as nucleic acids and proteins, is a reliable indicator of membrane damage induced by antimicrobial agents (Czajka et al., 2023; Di Rosario et al., 2025). In line with literature, PPE treatment caused a significant, dose-dependent leakage of dsDNA and proteins from yeast cells, confirming its ability to compromise membrane integrity. The effect was more pronounced in *C. glabrata* and *C. parapsilosis*, whereas the urogenital *C. albicans* isolate showed lower leakage levels, indicating greater resistance. These differences likely reflect species-specific variations in cell wall and membrane composition that influence susceptibility to PPE. The observed damage may result from the interaction of PPE polyphenols with membrane lipids, proteins, and ergosterol, leading to structural alterations and increased permeability, which in turn cause cytoplasmic leakage and the loss of essential cellular components (Sasidharan et al., 2023). These results suggest that PPE exerts its antifungal action primarily through the disruption of fungal membrane integrity and support its potential as a natural antifungal agent, particularly against non-*albicans Candida* species involved in vaginal infections. While membrane disruption appears to be the primary mechanism, metabolic targets cannot be excluded. Recent studies have highlighted several key enzymes in central metabolic pathways as potential antifungal targets, including acetyl-CoA synthetase (Acs), trehalose-6-phosphate synthase, and glucosamine-6-phosphate synthase, which are involved in energy metabolism and virulence in *Candida* species (Jezewski et al., 2025). These findings suggest that PPE may also interact with these metabolic pathways, warranting further investigation.

Antigerminative assays revealed a clear dose-dependent inhibition of germ tube formation by PPE against all tested *Candida* isolates. In both *C. albicans* and *C. parapsilosis*, significant inhibition occurred at concentrations of 20 μg μL^−1^, with near-complete suppression at 40 μg μL^−1^ (Figures 3A, C). In contrast, *C. glabrata*, which displayed a naturally lower capacity for germ tube formation, showed a marked reduction in germinative cells already at 10 μg μL^−1^ (Figure 3B). The MGIC values, reflecting the standardized antigerminative activity, were 40 μg μL^−1^ for all isolates treated with PPE (Table 4). In comparison, MGIC values obtained for tioconazole, used as a positive control (1.0 μg μL^−1^ for *C. albicans* and 0.5 μg μL^−1^ for *C. glabrata* and *C. parapsilosis*, Table 4), are generally considered cytotoxic. These findings highlight PPE not only as an effective inhibitor of germ tube formation but also as a potentially safer and more clinically promising alternative. To the best of our knowledge, no prior studies have performed a time-course germination inhibition assay using increasing concentrations of 50% hydroethanolic pomegranate peel extract on vaginal *Candida* strains. Even if the antigerminative effects of *Punica granatum* extracts have not been extensively explored in the literature, recent studies have shown that polyphenol-rich plant extracts can interfere with fungal morphogenetic transitions by compromising cell wall integrity and disrupting hyphal signaling pathways (Endo et al., 2010; Al-Madboly et al., 2022). The use of FITC-ConA fluorescence staining in this study further confirmed PPE's ability to inhibit hyphal development at 40 μg μL^−1^ PPE concentration (Figure 4), underscoring its potential in suppressing the formation of pathogenic morphotypes. These findings highlight the role of PPE not only in suppressing fungal proliferation but also in targeting critical virulence factors such as germ tube formation, particularly relevant in the pathogenicity of *Candida* species. Recent findings suggest that the bioactive compounds in pomegranate peel extract (PPE), particularly polyphenols such as punicalagin, ellagic acid, gallic acid, and other ellagitannins, exert antifungal effects through multiple molecular and metabolic mechanisms. Notably, one such mechanism involves the inhibition of glucose transporters, including HGT1 and HGT4, which has been proposed as a strategy to suppress fungal growth and biofilm formation (Chen et al., 2020).

**Table 4:** Antigerminative activity of hydroethanolic PPE and tioconazole against clinical isolates of *Candida* spp.

**Antifungal agent**	**MGIC (**μ**g** μ**L**^**−1**^**)**		
	***Candida albicans*** **UGPCA25**	***Candida glabrata*** **UGPCG25**	***Candida parapsilosis*** **UGPCP25**
PPE	40^****^	40^****^	40^****^
TCZ	1.0	0.5	0.5

Statistical significance was assessed using Student's *t*-test to compare the activity of PPE and tioconazole for each *Candida* isolate; asterisks indicate significance (^****^p < 0.0001). PPE, pomegranate peel extract; TZC, tioconazole; MGIC, minimum germ tube inhibition concentration.

The antigerminative activity may be linked to the disruption of key signaling cascades responsible for morphogenesis, such as the Ras1-cAMP-PKA-Efg1 pathway, which governs the yeast-to-hypha transition critical for *Candida albicans* virulence (Biswas et al., 2007). PPE compounds have been shown to alter membrane integrity and permeability, induce oxidative stress via reactive oxygen species (ROS) generation, and impair mitochondrial function, all of which may inhibit germ tube formation and reduce fungal viability (Endo et al., 2010).

Finally, in this study a significant antibiofilm activity of PPE was observed, highlighting its effectiveness against both the initial adhesion and maturation stages of *Candida* biofilms. Specifically, PPE demonstrated a dose-dependent reduction in biofilm biomass across all tested isolates, with the MBIC identified as 60 μg μL^−1^ for *C. albicans* (Table 3 and Figure 5A) and a notably lower MBIC of 20 μg μL^−1^ for *C. glabrata* and *C. parapsilosis* (Table 3 and Figures 5B, C). Importantly, these MBIC values are particularly significant when compared with those of the tioconazole, whose reported MBIC values against *Candida* biofilms are in the range of approximately 1 μg μL^−1^, concentrations that are generally considered cytotoxic for host tissues (Pierce et al., 2017; Silva et al., 2020). SEM analysis further confirmed these quantitative results by showing significant disruption of biofilm architecture on PVC surfaces at MBIC concentrations (Figure 6). Given the widespread use of PVC in medical devices and implants, these findings are particularly relevant for clinical contexts where biofilm-associated infections pose a serious threat. Notably, treatment with PPE led to marked degradation of the exopolysaccharide matrix, compromised fungal cell integrity, and significantly reduced adhesion to the PVC substrate. These morphological alterations indicate that PPE effectively compromises the key structural component required for biofilm stability and persistence on PVC surfaces. Such effects align with the known antifungal mechanisms of polyphenol-rich extracts, which interfere with biofilm matrix production and cell membrane integrity (Silva et al., 2020; Ferreira et al., 2025). These observations underscore the potential of PPE to inhibit biofilm formation on clinically relevant materials, an essential feature for addressing persistent and device-related *Candida* infections. Interestingly, PPE was also effective against mature biofilms, achieving eradication rates exceeding 60% in all isolates at concentrations threefold above the MBIC (Figure 7). This ability to disrupt established biofilms is particularly relevant given the clinical challenges posed by biofilm-associated resistance to conventional antifungals (Finkel and Mitchell, 2011), especially considering the MBEC values of tioconazole reported in Table 3. Notably, the MBEC of tioconazole against *Candida* biofilms reaches concentrations far exceeding physiologically tolerable levels. In contrast, PPE demonstrates potent antibiofilm activity at substantially lower and non-toxic concentrations, thereby representing a promising therapeutic alternative. In addition, the higher antibiofilm efficacy observed compared to prior studies, such as Fernandes et al. (2022) which reported a maximum 40% biofilm reduction in *C. albicans*, may be attributable to the high concentration of punicalagin and other polyphenols in our extract. Authors' previous analyses confirmed the presence of bioactive phenolic compounds in PPE (Pagliarulo et al., 2016). The Folin-Ciocalteu assay revealed a high total polyphenol content, exceeding 50 mg of gallic acid equivalents per milliliter of extract (mg GAE mL^−1^), while detailed phenolic profiling performed via reversed-phase high-performance liquid chromatography with diode-array detection (RP-HPLC-DAD) identified its major polyphenolic constituents, including punicalagin, pedunculagin, and ellagic acid. As reported in the literature, these molecules act synergistically to compromise fungal cell membrane integrity, reduce ergosterol levels, induce cell cycle arrest, thereby inhibiting biofilm formation and stability; additionally, they can interfere with quorum sensing and extracellular matrix synthesis, weakening the biofilm's structure, resilience, and pathogenicity (Silva et al., 2020). In detail, PPE can interfere with multiple stages of biofilm development, including initial adhesion, extracellular matrix (ECM) synthesis, and biofilm maturation. Some polyphenols have been demonstrated to downregulate the expression of biofilm-related genes such as ALS3, HWP1, BCR1, and EFG1, which are essential for adhesion, hyphal development, and ECM production (Nobile and Mitchell, 2005; Khan et al., 2023). In addition, the antioxidant and metal-chelating properties of PPE constituents may impair metal-dependent enzymes involved in biofilm structure and stability, thereby destabilizing the architecture of mature biofilms (Guevara-Lora et al., 2020; Er-rahmani et al., 2024). The synergistic interaction between these bioactive compounds likely contributes to the broad-spectrum antibiofilm and antigermination activity observed, even against non-*albicans Candida* species such as *C. glabrata* and *C. parapsilosis*. ‘Although further targeted molecular and metabolic studies are required, the existing evidence strongly supports the potential of PPE as a multifaceted antifungal agent.

## 5 Conclusions

Given the escalating challenge of antifungal resistance, especially in the context of biofilm-associated infections, this study provides evidence of the multifactorial *in vitro* efficacy of PPE against both established and emerging *Candida* species. The inhibitory effects observed on planktonic growth, morphogenetic transitions such as germ tube formation, and biofilm development highlight PPE's potential as a natural antifungal agent or as a bioactive coating to mitigate fungal colonization on medical devices. Although our study is limited to a small number of strains, it provides valuable insights into the antifungal, antigerminative, and antibiofilm activity of PPE against three clinically relevant *Candida* species, each exhibiting distinct susceptibility profiles to conventional azoles. These observations are consistent with previous studies that have reported meaningful results based on a limited number of isolates, including single-strain models, underscoring the utility of such preliminary analyses in generating foundational data for future research (Shaban et al., 2020; Fernández-Calderón et al., 2021; Parker et al., 2022). The antifungal efficacy of PPE appears to be mediated by its rich phytochemical composition, including polyphenols such as punicalagin, ellagic acid, and gallic acid, which may act synergistically to disrupt cell membrane integrity, interfere with morphogenetic signaling pathways, and compromise biofilm structure. However, the complex phytochemical nature of PPE necessitates comprehensive mechanistic investigations to elucidate the specific molecular targets and pathways involved. Investigating the effects of the tested pomegranate peel extract on both primary metabolism (e.g., carbon fluxes, ATP production) and secondary metabolism (e.g., mycotoxin-like compounds, candidolisins) could represents a promising in future for mechanistic investigations of metabolic perturbations. However, the complex phytochemical composition of pomegranate peel extract (PPE) necessitates comprehensive mechanistic studies to elucidate its specific molecular targets and the metabolic pathways involved. In particular, primary metabolism (e.g., carbon metabolism and ATP production) is essential for cellular vitality and may be a potential target of action for punicalagin and ellagic acid (Fahmy and Farag, 2022; Singh et al., 2023), while secondary metabolism (e.g., the production of gliotoxin-like compounds and candidolisins) is closely linked to the pathogenicity of emerging *Candida* species (Al-Dahlaki and Al, 2023; Ferreira et al., 2025). Therefore, investigating the effects of PPE on these metabolic processes represents a promising research direction for future experiments aimed at clarifying the mechanistic basis of its antifungal activity. Furthermore, rigorous *in vivo* studies are essential to validate the translational relevance of these findings, assess pharmacokinetics and toxicity profiles, and ultimately determine the feasibility of clinical application. Our results contribute to the growing body of knowledge on plant-derived antifungals and provide a rationale for future investigations involving a larger and more diverse set of clinical isolates. Importantly, the non-cytotoxic nature of the effective PPE concentrations further supports its potential for safe application. Future studies should focus on *in vivo* validation, pharmacokinetic profiling, and comprehensive safety assessment to evaluate the translational feasibility of PPE as an alternative or complementary antifungal strategy.

## Data Availability

The original contributions presented in the study are included in the article/Supplementary material, further inquiries can be directed to the corresponding authors.

## Supplementary material

Supplementary Figure S1 In vitro susceptibility of Candida vaginal isolates to standard azoles.

Supplementary Table S1 In vitro susceptibility of Candida vaginal isolates to standard azoles.
