# Supplementary material for: Antibiofilm sustainable strategies: pomegranate extract from agri-food waste as a natural antifungal against emerging Candida pathogens
Source: Front Microbiol. 2025 Nov 19;16:1724685. doi: 10.3389/fmicb.2025.1724685 (PMC12672473; doi:10.3389/fmicb.2025.1724685)
Supplement: Supplementary Figure S1 — In vitro susceptibility of Candida vaginal isolates to standard azoles. [file Data_Sheet_1.pdf]

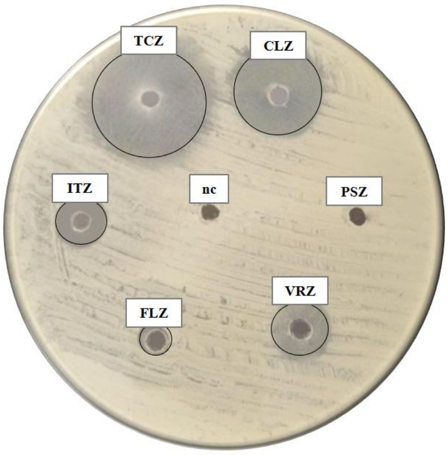

Candida albicans **UGPCA25**

Azole (1 mg/disc)	MDIZ (mm)
CLZ	18.33 ± 1.25
FLZ	7.76 ± 0.47
ITZ	10.33 ± 0.47
PSZ	0.00 ± 0.00
TCZ	23.00 ± 7.26
VRZ	11.67 ± 1.25

A)

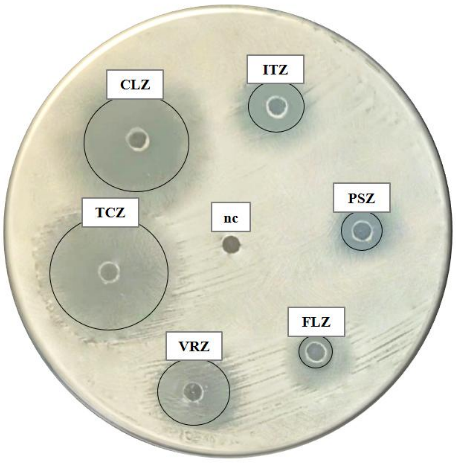

Candida glabrata **UGPCG25**

Azole (1 mg/disc)	MDIZ (mm)
CLZ	25.00 ± 0.82
FLZ	7.83 ± 0.24
ITZ	14.00 ± 0.82
PSZ	9.00 ± 0.82
TCZ	27.67 ± 1.25
VRZ	17.17 ± 0.62

B)

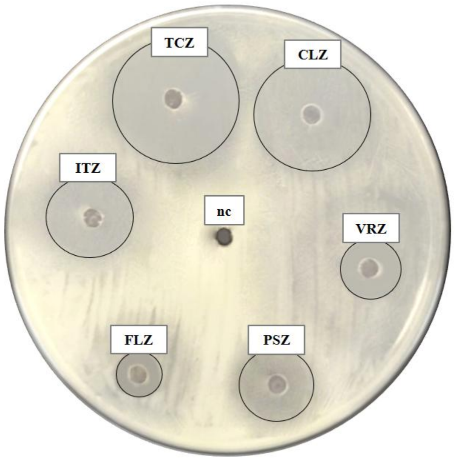

Candida parapsilosis **UGPCP25**

Azole (1 mg/disc)	MDIZ (mm)
CLZ	26.00 ± 0.82
FLZ	12.33 ± 0.47
ITZ	21.67 ± 0.47
PSZ	18.67 ± 0.94
TCZ	26.67 ± 3.77
VRZ	16.00 ± 0.82

C)

Figure S1. *In vitro* susceptibility of vaginal isolates *Candida albicans* UGPCA25 (A), *C. glabrata* UGPCG25 (B), and *C. parapsilosis* UGPCP25 (C) to standard azoles, evaluated using the agar well diffusion method. The mean diameter of inhibition zones (MDIZ, mm) was determined from triplicate assays and is expressed as mean ± standard deviation, reflecting the antifungal activity of each azole (1 mg/disc). CLZ, climbazole; FLZ, fluconazole; ITZ, itraconazole; PSZ, posaconazole; TCZ, tioconazole; VRZ, voriconazole; nc, negative control.

Table S1. Susceptibility profile of vaginal isolates *Candida albicans* UGPCA25, *C. glabrata* UGPCG25, and *C. parapsilosis* UGPCP25 to standard azoles.

**Azole**	**MIC (µg µL^{-1})**		
	C. albicans **UGPCA25**	***C. glabrata*** **UGPCG25**	***C. parapsilosis*** **UGPCP25**
**CLZ**	0.80 (N/A[#])	0.60 (N/A[#])	0.40 (N/A[#])
**FLZ**	2.00 (S[#])	4.00 (R[#])	0.50 (S[#])
**ITZ**	1.00 (R[#])	0.60 (R[#])	0.20 (R[#])
**PSZ**	> 4.00 (R[#])	> 4.00 (R[#])	0.06 (S[#])
**TCZ**	0.20 (N/A[#])	0.10 (N/A[#])	0.10 (N/A[#])
**VRZ**	0.80 (R[#])	0.20 (R[#])	0.20 (R[#])

Minimum inhibitory concentrations (MICs, µg/mL) were determined by broth microdilution according to CLSI (Clinical and Laboratory Standards Institute) guidelines. CLZ, climbazole; FLZ, fluconazole; ITZ, itraconazole; PSZ, posaconazole; TCZ, tioconazole; VRZ, voriconazole; na, not applicable. [#] Susceptibility categories (S = susceptible, R = resistant) are indicated in round brackets next to each MIC value and interpreted, where applicable, according to EUCAST (European Committee on Antimicrobial Susceptibility Testing) clinical breakpoints v.11.0 (https://www.eucast.org/clinical_breakpoints/); N/A, not applicable.
